# Long-Term Disease-Free Survival Without Radiotherapy in a Pediatric Patient With Neurofibromatosis Type 1-Associated Medulloblastoma: A Case Report

**DOI:** 10.7759/cureus.94302

**Published:** 2025-10-10

**Authors:** Iori Ozono, Fumiyuki Yamasaki, Shumpei Onishi, Ushio Yonezawa, Nobutaka Horie

**Affiliations:** 1 Neurosurgery, Hiroshima University Hospital, Hiroshima, JPN; 2 Neurosurgery, HIroshima University Hospital, Hiroshima, JPN

**Keywords:** autologous stem-cell transplantation, chemotherapy, neurofibromatosis type 1 (nf-1), pediatric cns neoplasm, pediatric medulloblastoma

## Abstract

Neurofibromatosis type 1 (NF1) is an autosomal-dominant disorder associated with an increased risk of central nervous system tumors, particularly low-grade gliomas. However, the development of medulloblastoma in NF1 patients is extremely rare. Given the heightened risk of secondary malignancies following radiotherapy in this population, treatment strategies that minimize radiation exposure are important. We report a case of a two-year-old boy with a family history of NF1 who presented with headache and ataxia. Brain MRI revealed a 45-mm mass in the cerebellar vermis. Gross total resection was performed, and histopathology confirmed desmoplastic/nodular medulloblastoma. Postoperatively, the patient received multi-agent chemotherapy and intrathecal methotrexate without adjuvant radiotherapy. Consolidation therapy included high-dose chemotherapy with etoposide, carboplatin, and melphalan, followed by autologous peripheral blood stem cell transplantation. The treatment was completed successfully despite some toxicities. More than 10 years after treatment, the patient remains in complete remission without evidence of recurrence or late complications. This case highlights the potential of radiation-free, individualized treatment strategies for patients with NF1 to minimize the risk of secondary malignancies and achieve favorable long-term outcomes.

## Introduction

Neurofibromatosis type 1 (NF1) is an autosomal-dominant inherited disorder with an estimated prevalence of 1 in 3,000 individuals and without significant racial variation [1]. More than half of all NF1 cases arise sporadically due to de novo mutations [2]. The condition presents with various symptoms, including café-au-lait spots (light brown pigmented spots on the skin), neurofibromas, freckling, Lisch nodules (pigmented nodules in the iris), and bone deformities, all of which contribute to the diagnostic criteria [3]. Patients with NF1 have an increased risk of developing central nervous system (CNS) tumors, particularly in pediatric cases, where approximately 20% of all patients are affected [4]. The most common CNS tumors in these patients are low-grade gliomas, such as optic pathway and brainstem gliomas [5,6]. Although NF1 is recognized as a cancer predisposition syndrome (CPS), meaning that patients have a higher lifetime risk of developing cancer due to underlying genetic factors, the development of medulloblastoma is extremely rare [7]. Gross total resection is a well-established positive prognostic factor in medulloblastoma [8], and standard postoperative treatment typically involves a combination of chemotherapy and radiation therapy [9]. However, patients with CPS are at an increased risk of developing secondary malignancies, particularly after radiotherapy [10]. Reports of medulloblastoma in patients with NF1 are extremely rare, with only 10 cases documented to date [7,11-18]. In this report, we present a case of medulloblastoma associated with NF1 that was successfully treated with chemotherapy alone. This case highlights the clinical importance of radiation-sparing strategies not only to reduce the risk of secondary cancers but also to preserve neurocognitive function and long-term quality of life in this vulnerable population.

## Case presentation

**Figure 1:**
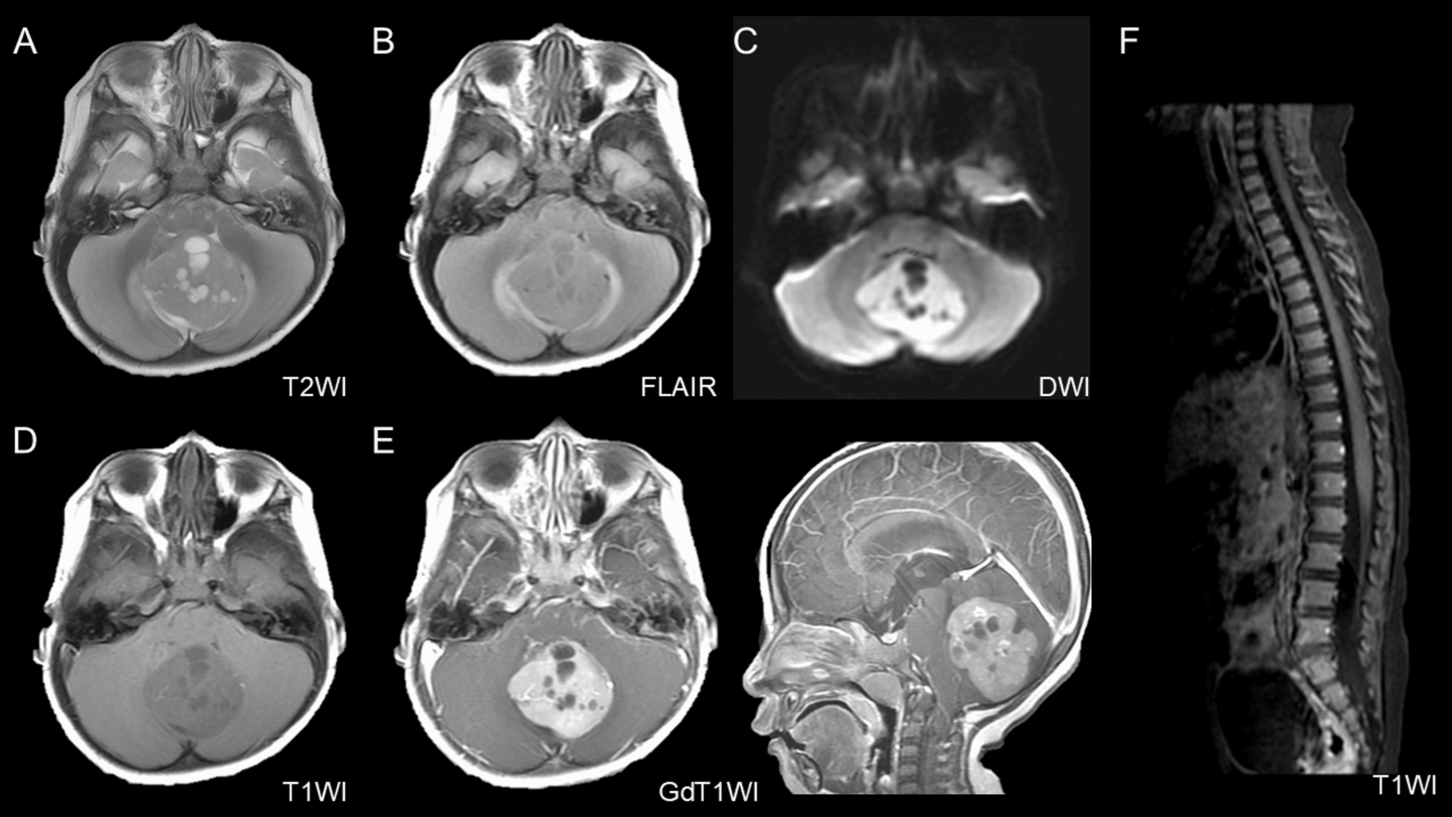
Preoperative brain MRI showing a posterior fossa tumor. A 45-mm tumor was observed in the fourth ventricle. The tumor shows multiple cystic components on T2-weighted imaging (T2WI) (A) and peritumoral edema on fluid-attenuated inversion recovery imaging (FLAIR) (B). It appears hyperintense on diffusion-weighted imaging (DWI) (C). On plane T1-weighted imaging (T1WI) (D), the tumor appears slightly hypointense and exhibits homogeneous enhancement after gadolinium administration (E). T1-weighted imaging showed no evidence of spinal dissemination (F).

A two-year-old boy with a family history of NF1 in his mother and maternal grandmother was being followed up by our dermatology department for multiple café-au-lait spots on his trunk and extremities, consistent with a clinical diagnosis of NF1. The patient presented with headache and ataxia. Brain magnetic resonance imaging (MRI) revealed a 45-mm tumor located in the cerebellar vermis and extending into the fourth ventricle, causing obstructive hydrocephalus (Figure 1).

**Figure 2:**
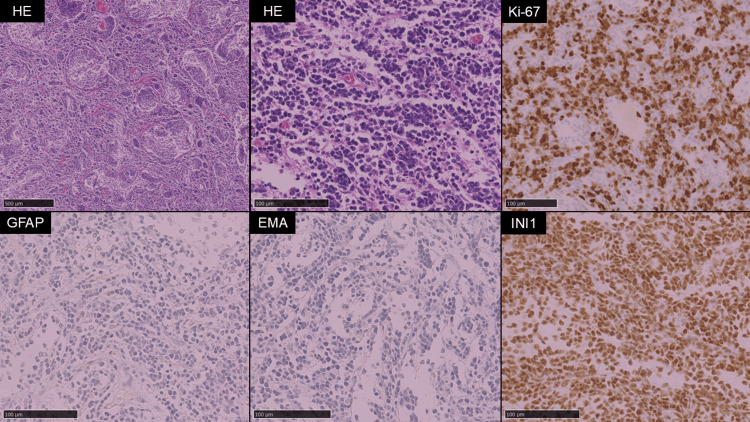
Histological features of the resected cerebellar tumor. Hematoxylin and eosin (HE) staining revealed nodular structures lacking reticulin fiber formation, surrounded by densely reticulated areas with proliferation of poorly differentiated tumor cells. The Ki-67 labeling index was approximately 80% in the internodular regions. Immunohistochemical analysis revealed that the tumor cells were negative for glial fibrillary acidic protein (GFAP) and epithelial membrane antigen (EMA), whereas integrase interactor 1 (INI-1) expression was retained.

The tumor exhibited multiple cystic components and demonstrated homogeneous contrast enhancement with well-demarcated margins from the surrounding brain tissue. No spinal lesions were identified. A gross total resection of the tumor was achieved via a midline suboccipital craniotomy. The perioperative course was uneventful, with no intraoperative complications or new neurological deficits observed, allowing timely initiation of adjuvant chemotherapy. Histopathological examination (Figure 2) revealed pale, nodular structures lacking reticulin fiber formation, surrounded by densely reticulated areas with proliferation of poorly differentiated tumor cells. The Ki-67 labeling index was approximately 80% in the internodular regions. Immunohistochemistry revealed that the tumor cells were negative for glial fibrillary acidic protein (GFAP) and epithelial membrane antigen (EMA), whereas integrase interactor 1 (INI-1) expression was retained. These findings are consistent with desmoplastic/nodular medulloblastoma.

**Figure 3:**
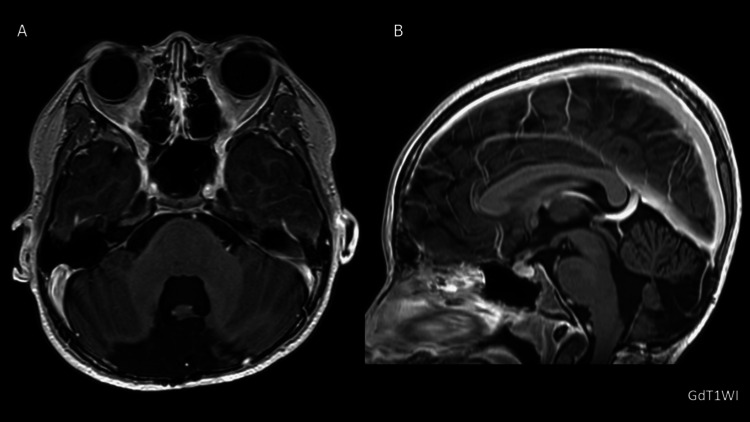
Long-term follow-up MRI after chemotherapy and stem cell transplantation. (A, B) A follow-up gadolinium-enhanced T1-weighted MRI obtained more than 10 years after treatment revealed no evidence of medulloblastoma recurrence.

Following surgery, the patient underwent five cycles of multi-agent chemotherapy comprising ifosfamide/cyclophosphamide, etoposide, vincristine, and carboplatin/cisplatin. Intrathecal methotrexate was administered during cycles 2-5. The selected regimen, consisting of platinum compounds, alkylating agents, vincristine, etoposide, and intrathecal methotrexate, was based on national protocols in use at the time and is consistent with the Japanese Practical Guidelines for Neuro-Oncology, pediatric edition 2022 [19], which recommend multi-agent chemotherapy of this composition for patients younger than three years with desmoplastic/nodular medulloblastoma to delay or avoid craniospinal irradiation. Treatment-related toxicities included grade 3 elevations in aspartate aminotransferase and alanine aminotransferase, as well as grade 4 neutropenia, according to the Common Terminology Criteria for Adverse Events (CTCAE). Despite these adverse events, all planned chemotherapy courses were completed. Consolidation radiotherapy was deliberately avoided. The decision to omit radiotherapy was based on both the patient’s very young age and the presence of NF1, considering the increased risk of cognitive impairment and secondary malignancies in this population. Instead, the patient received high-dose chemotherapy with etoposide, carboplatin, and melphalan, followed by autologous peripheral blood stem cell transplantation (auto-PBSCT). Engraftment was confirmed on day 9 after transplantation. The only significant complication during this phase was CTCAE grade 3 oral mucositis, which was managed with temporary nasogastric tube feeding. The patient was discharged on day 65. More than 10 years after surgery, the patient remains in complete remission, with no evidence of tumor recurrence or long-term treatment-related sequelae (Figure 3). While there are no major neurological deficits, the patient has experienced learning difficulties beginning in junior high school and now attends a special-needs class for academic support. This finding underscores the importance of considering long-term neurocognitive outcomes even in radiation-free treatment strategies.

## Discussion

To date, only 10 cases of medulloblastoma in patients with NF1 have been reported, highlighting the extreme rarity of this association (Table 1) [7,11-18]. To the best of our knowledge, this is the first reported case of a patient with NF1 and medulloblastoma treated with a radiotherapy-sparing strategy that included high-dose chemotherapy, followed by auto-PBSCT. The patient remained disease-free for more than 10 years, with no evidence of recurrence or radiation-associated complications, highlighting the potential long-term benefits of avoiding radiotherapy in this vulnerable population.

**Table 1:** Reported cases of medulloblastoma in patients with neurofibromatosis type 1. CR: complete remission; N/A: not available; MB: medulloblastoma

Authors	Published year	Age	Histology	Radiotherapy	Outcome	Follow-up
Corkill and Ross [11]	1969	8	N/A	2975r	Died at 17 years because of metastatic sarcomatous nerve tumors in the MB CR	9 years
Perilongo et al. [12]	1993	8	N/A	N/A	N/A	N/A
Martínez-Lage et al. [13]	2002	6	Classic	Posterior fossa 40Gy; Craniospinal axis 30Gy	Alive in CR	5 years
Rosenfeld et al. [14]	2010	16	Anaplastic	Posterior fossa 57.6Gy; Craniospinal 36Gy	Alive in CR	11 months
Pascual-Castrovejo et al. [15]	2010	4	N/A	Performed	Died in seven years	3 years
Varan et al. [16]	2015	4	N/A	N/A	Died in four years	1 month
	2015	9	N/A	N/A	Alive in CR	11 months
Vanan et al. [17]	2016	N/A	N/A	N/A	Alive in CR	6 months
Marinău et al. [18]	2017	4	Desmoplastic	None	Died in four years	6 months
Ranalli, et al. [7]	2021	14	Classic	None	Alive in CR	1 year
Ozono et al. (present case)	2025	2	Desmoplastic/nodular	None	Alive in CR	11 years

Among the 10 reported cases, four patients received postoperative radiotherapy. In one notable case, an eight-year-old boy underwent surgical resection of a medulloblastoma located in the right cerebellar hemisphere, followed by postoperative radiotherapy totaling approximately 26 Gy (2,975 R). The patient died nine years after disease onset, and autopsy findings revealed a sarcomatous neurofibroma and thyroid carcinoma, which were presumed to be radiation-induced complications [11]. More recently, in 2021, a 14-year-old patient, similar to our case, was treated with multi-agent chemotherapy alone because of concerns regarding secondary malignancies associated with radiation and has progressed without recurrence, albeit for a short period of one year [7]. Our treatment decision to omit radiotherapy was consistent with contemporary consensus for children younger than three years with medulloblastoma. The SJYC07 trial, a multicenter, phase 2 study, demonstrated that risk-adapted chemotherapy-alone regimens can achieve favorable long-term survival in infants and young children with desmoplastic/nodular or Sonic Hedgehog (SHH)-activated medulloblastoma without metastasis, while sparing them from the neurocognitive sequelae of craniospinal irradiation [20]. In our case, the patient was treated in 2015, when a consensus had already been reached to delay or avoid radiotherapy in this population due to concerns about developmental and cognitive outcomes. The presence of NF1 provided an additional rationale for radiation avoidance, given the elevated risk of secondary malignancies after irradiation. Thus, both factors, age-related neurocognitive risk and NF1 status, were considered in our decision-making process.

Among the risks of benign and malignant tumors associated with NF1 [21], the development of secondary malignancies following radiation therapy has become a subject of increasing clinical concern. In a study comparing patients with NF1 and optic pathway gliomas who received radiation therapy with those who did not, the relative risk of developing secondary malignancies was 3.04 (95% confidence interval (CI): 1.29-7.15) in the irradiated group [10]. Reflecting this elevated long-term risk, the Children’s Oncology Group Long-Term Follow-Up Guidelines recommend a brain MRI every other year beginning two years after radiation therapy in patients with NF1 [22]. Beyond NF1, data from medulloblastoma cohorts also demonstrate a markedly increased risk: the observed-to-expected ratio of secondary malignant neoplasms was 4.49 (95% CI: 3.53-5.62), with a particularly elevated ratio in the CNS (40.62; 95% CI: 25.46-61.51).

Several factors may have contributed to the favorable long-term survival observed in this case. First, gross total resection was achieved, which is a well-established positive prognostic indicator in medulloblastoma. Second, no evidence of spinal dissemination was found, further reducing the risk of recurrence. Third, the patient was able to complete the planned multi-agent chemotherapy regimen, which likely enhanced disease control. In addition, although glioblastoma represents a distinct clinical entity, patients with glioblastoma associated with NF1 have been reported to have better prognoses than those without NF1 [23]. This observation raises the possibility that NF1 status could potentially influence prognosis in medulloblastoma. Taken together, these clinical and biological factors may explain the survival time exceeding 10 years without the use of radiotherapy, supporting the importance of considering radiotherapy-sparing strategies in NF1-associated cases whenever possible.

A limitation of this report is that we cannot definitively prove that the medulloblastoma was directly caused by the NF1 germline mutation rather than representing an unrelated second primary tumor. While the clinical and family history support the diagnosis of NF1, definitive proof of NF1-driven tumorigenesis would require genetic confirmation of a germline NF1 pathogenic variant and demonstration of biallelic inactivation in the tumor. Such a two-hit pattern is well-documented in NF1-associated tumors and would provide strong evidence of causality. In the absence of this data, we cautiously describe the lesion as a medulloblastoma occurring in a patient with NF1. Future work could include germline NF1 testing, tumor sequencing, and methylation-based subgrouping to clarify the genetic basis of this association. Another limitation is the lack of comparative data on outcomes between NF1-associated and non-NF1 medulloblastoma patients treated with chemotherapy alone. To our knowledge, no study has directly addressed this question, and therefore, the prognostic implications of NF1 status in this setting remain uncertain.

## Conclusions

This case of medulloblastoma in a pediatric patient with NF1 was managed without radiotherapy, resulting in more than 10 years of disease-free survival. In very young NF-1 patients with medulloblastoma who cannot receive radiation therapy, surgical resection and chemotherapy remain the major treatments. Long-term interval neuroimaging is mandatory for the detection of recurrent tumors or consideration of subsequent radiation therapy. If there is no postoperative tumor recurrence, avoidance of radiation therapy is an option for preventing radiation-induced adverse effects or second malignant tumors. Further studies are needed to clarify the optimal management strategies for NF1-associated medulloblastoma.
